# Skin Assessment Strategies for Identification of Pressure Injuries in Dark-Skin-Tone Patients: A Scoping Review

**DOI:** 10.3390/healthcare14162655

**Published:** 2026-08-21

**Authors:** Vainess Banda Mbuzi, Susan Morgan, Dianne Stratton-Maher, Tracey Tulleners, Leah East

**Affiliations:** School of Nursing and Midwifery, University of Southern Queensland, Ipswich, QLD 4305, Australia; sue.morgan@unisq.edu.au (S.M.); dianne.stratton-maher@unisq.edu.au (D.S.-M.); tracey.tulleners@unisq.edu.au (T.T.); leah.east@unisq.edu.au (L.E.)

**Keywords:** dark skin, skin tone, skin assessment strategies, pressure injury

## Abstract

**Highlights:**

**What are the main findings?**

- Ten studies identified four assessment categories: thermography, SEM sensors, image processing/colorimetry, and enhanced lighting.
- Objective technologies consistently detected early tissue changes before visual signs appeared.
- These technologies maintained diagnostic performance across diverse skin tones, including dark skin tones.
- Routine visual and tactile assessment was underrepresented and shown to be unreliable for early detection in darker skin.
- No study evaluated a multimodal assessment approach, revealing a clear evidence gap.

**What are the implications of the main findings?**

- Clinical practice must reduce reliance on visual inspection, which lacks sensitivity in richly pigmented skin.
- Integrating objective technologies into routine care can improve diagnostic equity and early detection.
- Thermography, SEM, colorimetry, and enhanced lighting offer feasible pathways for more accurate assessment.
- Strengthening the evidence base for bedside assessment in dark skin tones is essential for safe, equitable care.

**Abstract:**

**Background:** Early pressure injury detection is essential in acute care, yet diagnostic inequities persist for individuals with dark skin tones. Traditional visual assessment relies on colour changes that may not be visible in richly pigmented skin, contributing to delayed diagnosis and poorer outcomes. **Objective:** Our aim was to identify and map strategies reported in the literature for assessing skin and underlying tissue to support early pressure injury detection in adults with dark skin tones in acute care settings. **Methods:** A scoping review was conducted following Joanna Briggs Institute methodology and reported according to PRISMA-ScR. Searches were performed in CINAHL, PubMed, Scopus, Web of Science, and Google Scholar (April 2025; updated February 2026). Primary research published in English since 2017 was eligible. After screening and data extraction, descriptive thematic analysis was applied to identify recurrent strategies reported in the literature for assessment of skin and underlying tissues for detection of pressure injury. No appraisal of the included sources was conducted. **Results:** Ten studies met the inclusion criteria. Strategies identified for detecting pressure injuries were mainly for implementation across diverse skin tones, but all included aspects of dark-skin-tone participants. These approaches are clustered into four categories: thermographic imaging, subepidermal moisture sensors, image processing and colorimetry, and enhanced assessment and lighting techniques. The evidence highlights the growing value of objective, technology-supported methods for improving detection in people with dark skin tones. Routine bedside visual and tactile assessment was notably underrepresented, indicating a clear evidence gap. Overall, the review emphasises the need to integrate objective technologies into everyday practice to support more equitable pressure injury detection. **Conclusions:** This scoping review highlighted that evidence for routine visual and tactile assessment in dark skin tones remains limited. Technology-supported assessment approaches show promise for improving diagnostic equity in pressure injury detection. Identification of strategies used to perform skin assessment for pressure injury identification is critical to the wellbeing of persons with dark skin tones. This review demonstrates that while emerging technologies offer promising ways to improve pressure injury detection across diverse skin tones, evidence supporting everyday bedside visual and tactile assessment for individuals with dark skin tones remains notably limited. Strengthening this evidence base and integrating objective technologies into routine care are essential steps toward more accurate and equitable pressure injury detection.

## 1. Introduction

Acute care settings represent high-risk clinical environments where patients frequently experience sudden immobility, acute physiological deterioration, and hemodynamic instability, all of which substantially increase their vulnerability to pressure injury (PI) development [1,2]. Pressure injuries, also referred to as pressure ulcers, bedsores or decubitus ulcers, occur when sustained pressure on localised areas leads to tissue damage, most commonly over bony prominences such as the sacrum, coccyx, heels, hips, knees and ankles [3]. PIs present a major risk to patient health outcomes, elevating the risk of infection, extending hospital stays, and increasing morbidity and mortality [4,5]. Despite decades of research identifying risk factors, informing prevention strategies, and establishing evidence-based treatment plans [3,6], PIs remain one of the most frequently occurring yet largely preventable complications in acute care and are considered a key indicator of quality of care [7]. Studies continue to emphasise the complex nature of PIs while reinforcing that they are largely preventable [8,9].

PIs are classified into four stages, ranging from intact skin with colour changes (stage one) to full-thickness tissue loss with exposed muscle, tendon or bone (stage four) [6]. Early identification is critical, as stage one injuries act as early warning signs of underlying tissue damage and are reversible if detected promptly [10]. Delayed recognition increases the risk of progression to deeper, more complex wounds, leading to extended hospital stays, increased treatment costs, and poorer patient outcomes, including premature death [11,12]. Clinicians therefore require strong assessment skills to detect early tissue changes and prevent deterioration [12,13].

Accurate skin assessment is essential for PI identification; however, early visual signs differ across skin tones [14]. Individuals with dark skin tones often do not display blanching, erythema, or the pink or purple hues typically used to identify early tissue damage in lighter skin tones [14,15,16]. Evidence shows that individuals with dark skin tones are at increased risk of delayed or missed diagnosis due to inadequate assessment techniques and tools historically developed around lighter skin pigmentation [13,17]. As a result, inequities persist, with higher rates of undetected stage one PIs and more advanced presentations reported among patients with darker skin tones [18]. Increasing global migration has resulted in diverse skin pigmentation patterns across patient populations [19,20], making it essential for clinicians to understand how skin tone influences PI presentation and adapt assessment practices accordingly [21,22].

Acute care settings such as medical/surgical wards, orthopaedics, emergency departments, operating theatres, and intensive care units are time-pressured and complex, with patients frequently presenting with comorbidities that further compound their risk [2]. In these environments, missed early signs of pressure injury can lead to rapid deterioration, making accurate and equitable assessment practices particularly critical. Research indicates that approximately 12.9% of hospital admissions in Australia and New Zealand have a pressure injury, while a further 7.9% develop one during their stay [23]. This study offers acute care-specific prevalence data that is relevant to the context of this review.

Although extensive literature exists on general pressure injury assessment, there is a clear gap in research specifically examining how clinicians identify early-stage pressure injuries in adults with dark skin tones [14,15,22]. Addressing this gap is essential to improving diagnostic accuracy, promoting equity, and preventing avoidable harm. Without targeted strategies, individuals with dark skin tones remain at risk of delayed identification and poorer clinical outcomes [17]. This scoping review contributes to ongoing efforts to advance health equity by mapping evidence on approaches that support earlier and more accurate detection. The findings provide direction for future research, guideline development, and evidence-based recommendations aimed at promoting inclusive, culturally safe, and effective pressure injury assessment for individuals with dark skin tones.

A preliminary search of MEDLINE, the Cochrane Database of Systematic Reviews and JBI Evidence Synthesis was conducted, and no current or underway systematic reviews or scoping reviews on the topic were identified.

## 2. Materials and Methods

### 2.1. Study Design

This scoping review followed the Joanna Briggs Institute (JBI) methodology and was reported according to Preferred Reporting Items for Systematic reviews and Meta-Analyses extension for scoping reviews (PRISMA-ScR) [24,25]. The JBI-recommended PCC (Participants, Concept, Context) framework for scoping reviews was used to guide the development of the research question and inclusion and exclusion criteria for this scoping review [24]. This scoping review was registered with the Open Science Framework (OSF) Registration DOI: 10.17605/OSF.IO/9BWN3).

### 2.2. Research Question

What strategies are used within acute care settings to assess and identify pressure injuries in adult patients with dark skin tones?

### 2.3. Eligibility Criteria

Participants

- Adults ≥ 18 years
- Identified as having dark skin tones
- Receiving care in acute care settings
- Studies with mixed skin tones which include dark-skin-tone participants

Mixed-tone samples were included only when findings were stratified or discussed in relation to darker skin tones.

Concept:

Strategies, tools, or technologies used to assess skin or underlying tissue for pressure injury detection.

Context:

Acute care settings include but not limited to emergency departments, intensive care units, medical/surgical wards, and operating theatres.

Exclusions:

- Studies focused solely on prevention or treatment;
- Non-primary research, guidelines, best practice documents, opinion papers;
- Non-acute settings.

### 2.4. Search Strategy

This scoping review utilised a three-step approach to identify published studies. An initial search of Medline (Web of Science) and CINAHL Ultimate (EBSCOhost) was undertaken to identify descriptive terms. A full search strategy was developed from the keywords and index terms compiled from the initial search. The search strategy incorporated the Boolean operators Medical Subject Headings (MeSH) and text words in various combinations. The search strategy followed the Population, Concept, Context framework:

Concept 1 (“Skin Pigmentation”[Mesh] OR “higher melanin” OR “ethnic skin” OR “skin colour” OR “skin color” OR “skin tone*” OR “dark skin” OR “darker skin” OR “skin pigmentation*” OR “diverse skin” OR Ethnicity OR race), AND Concept 2 (“Pressure Ulcer”[Mesh] OR “pressure injur*” OR “bed sore*” OR Bedsore* OR “decubitus ulcer*” OR “decubitus sore*” OR “pressure ulcer*”), Concept 3 (“Skin Care”[Mesh] OR “skin integrity assessment*” OR “skin check*” OR “skin inspection*” OR “skin assessment*”), Concept 4 (Identif* OR Recognition OR isolating), AND Concept 5 (“Hospitals”[Mesh] OR “Patients’ Rooms”[Mesh] OR “Hospital Units”[Mesh] “acute care” OR Hospital* OR “intensive care” OR Ward OR wards). In consultation with the research librarian, Concepts 3, 4, and 5 were excluded from the final search strategy because their inclusion substantially restricted the number of retrieved records and risked omitting relevant studies.

Using Concept 1 and Concept 2 of the search strategy, a search of all included databases and information sources was conducted with the search strategy being adapted for each database and information sources. The online databases searched include CINAHL (EBSCOhost), Google Scholar, PubMed (EBSCOhost), Scopus (Elsevier), and Web of Science (See Appendix A). The original data search was conducted from 8 April to 10 April 2025 and was updated on 3 February 2026. No further studies were found from the updated search. In line with JBI guidance, scoping reviews are intentionally broad to ensure that all potentially relevant evidence is captured. Although this approach widened our search, the number of records retrieved was manageable, and the screening process was conducted systematically using our predefined protocol. This structured workflow ensured consistency, feasibility, and rigour throughout study selection.

Peer-reviewed studies published in English since 2017 were considered for inclusion. The scoping review was limited to English due to the linguistic abilities of the authors, and including studies published since 2017 ensured contemporary practice was described. This date range is also reflective of changes made in 2016 to the widely used National Ulcer Advisory Panel (NPUAP). These pressure injury classification system guidelines were updated to include dark skin pigmentation that may present differently [26].

### 2.5. Identifying Relevant Studies

**Figure 1:**
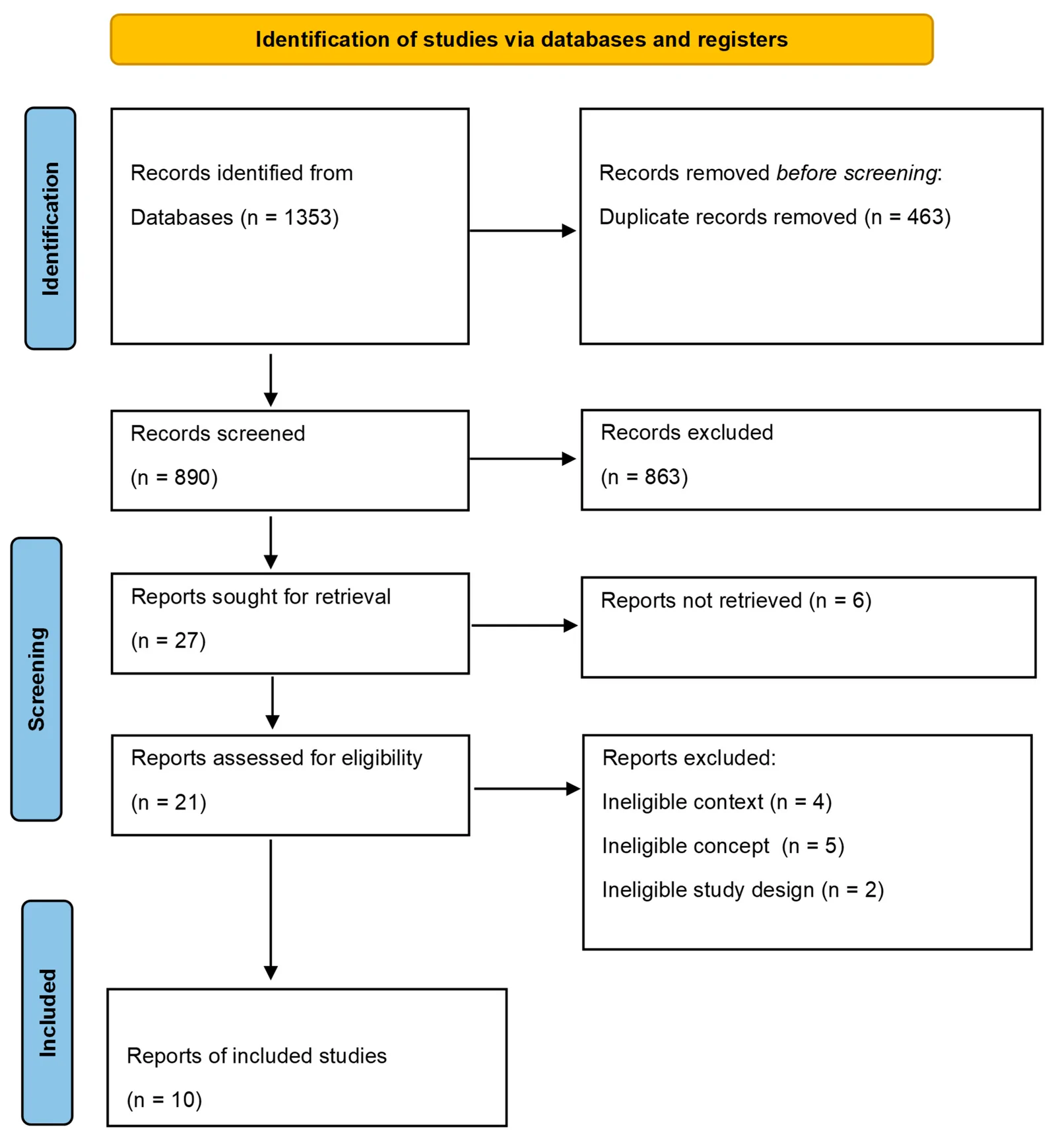
PRISMA-ScR flow diagram; Pressure injury and skin tone scoping review, 8 April to 10 April 2025. Source: Page MJ, et al., 2021 [28].

Following the database searches, all records identified were imported into EndNote 21.4 (author/reviewer 1) (Clavirate Analytics, PA, USA). Following duplicate removal, all retrieved records were uploaded to JBI System for Unified Management, Assessment and Review of Information (JBI SUMARI) [27]. The titles and abstracts of all retrieved records were screened by two independent reviewers to identify studies that potentially met the inclusion criteria. Full-text articles for all potentially relevant studies were then obtained and assessed in detail by two reviewers against the inclusion and exclusion criteria. Any disagreements regarding study eligibility were resolved through discussion or in consultation with a third reviewer. Reasons for exclusion at the full text review were recorded. A PRISMA flow diagram (see Figure 1) was employed to document the study inclusion process, and the results of the search are reported in the scoping review. This scoping review considered quantitative, qualitative and mixed methods studies for inclusion.

### 2.6. Data Extraction and Synthesis

Data was extracted by two independent reviewers using a data extraction chart designed for the review. The data extraction chart captured the study type, methodology, method, aim of the study, country, participant type and ages, setting, strategy or practice used to assess for pressure injury, and key findings. Any disagreements were resolved through discussion or with a third reviewer. As this is a scoping review, no critical appraisal of individual information sources was undertaken. The purpose was to map and explore information available in the literature [24].

Analysis adopted a descriptive thematic analysis approach to identify and categorise key findings from the studies [29]. Descriptive thematic analysis provides a flexible yet structured framework that can be modified to suit different study designs and data types [30]. Initially, the included studies were reviewed to extract similarities and patterns with these used to construct preliminary themes. The researchers individually read and reread the data and then compared and discussed the arising themes. Commonalities were synthesised, and a narrative summary was used to describe how the results have addressed the review objectives and questions. This was completed due to lack of homogeneity of strategies that were presented in the papers included.

## 3. Results

### 3.1. Study Selection

The original electronic database search yielded 1353 records. After removal of 463 duplicates, 890 records were screened by title and abstract. Of these, 863 were excluded as not meeting the eligibility criteria, and 27 records were sought for full text retrieval. Among the 27 records, 6 could not be retrieved because they were unavailable. The six reports not retrieved were items for which full-text access could not be obtained despite attempts via institutional access and interlibrary loan. The remaining 21 records were assessed for inclusion in the review, leading to exclusion of 11 records with the remaining 10 studies included in the scoping review. The reasons for exclusion were ineligible concept (*n* = 5), ineligible context (*n* = 4), and ineligible study design (not primary research) (*n* = 2). The full search was repeated on the 3rd of February 2026 to update and identify any additional studies since the original search. The updated search retrieved no additional studies to include in the review. The screening process used to select eligible studies is depicted in the PRISMA-ScR flow diagram [28] in Figure 1.

### 3.2. Study Characteristics

Collectively, the evidence base demonstrated substantial methodological diversity, reflecting the exploratory and practice-focused nature of the field. Study designs included case study (*n* = 1) [31]; pre-post experimental (*n* = 2) [32,33]; quality improvement projects using quantitative approaches (*n* = 2) [16,34]; non-experimental repeated-measures descriptive design (*n* = 1) [35]; quasi-experimental (*n* = 1) [36]; cross-sectional (*n* = 1) [37], retrospective, descriptive comparative study (*n* = 1) [38]; and a descriptive, prospective study (*n* = 1) [39]. Details of the characteristics of the included studies are indicated in Table 1.

All ten included studies were conducted in the United States. Sample sizes ranged from 3 to 140 participants, with varying representation of dark skin tones. Several studies included mixed-tone samples but reported findings relevant to darker skin tones, consistent with the PCC criteria. Across the ten included studies, a clear pattern emerged; while methodologies and technological approaches varied, each study sought to address the persistent challenge of detecting early pressure injuries in individuals with darker skin tones. Traditional visual assessment alone was repeatedly shown to be insufficient for identifying subtle, early-stage tissue changes in richly pigmented skin, prompting researchers to explore more objective, physiologically grounded, and technologically enhanced methods. A detailed summary of each study included is provided below.

### 3.3. Themes and Subthemes

An analysis of the included studies found one main overarching theme: the use of technology as a critical strategy for improving the early detection of pressure injuries in individuals with dark skin tones. Across all the studies, technology emerged not only as an adjunct to clinical assessment but as a necessary response to longstanding diagnostic inequities. These studies highlighted the fact that traditional visual assessment methods often fail to identify early skin changes in richly pigmented skin. In contrast, technological tools such as subepidermal scanners, thermography, digital imaging systems, appropriate light-enhanced assessment protocols, and appropriate lighting provided more objective indicators of early tissue damage.

Four sub-themes were identified within this framework:

1. Thermographic imaging;
2. Subepidermal moisture sensors;
3. Image processing algorithms and colorimetry;
4. Enhanced assessment and lighting assessment strategies.

#### 3.3.1. Thermographic Imaging

Thermographic imaging was used in three studies. Lam [31] reported that longwave infrared thermography identified thermal anomalies (>1.2 degrees ± variance) in patients with dark skin tones. Long-wave infrared thermography identified thermal anomalies before any visual signs of pressure injury, demonstrating early detection capability independent of skin pigmentation.

Bates-Jensen et al. [32] conducted a pre/post experimental study involving 61 participants across diverse skin tones (18 African American or Black; 16 Asian American; 23 White; 4 mixed race) to examine whether thermal measurement could support early identification of erythema. The study found that thermal patterns were consistent across all skin tones, demonstrating that infrared emissivity is tone-independent and therefore capable of detecting early tissue changes regardless of pigmentation. In contrast, visual erythema detection varied significantly by skin tone. Participants with lighter skin tones displayed greater measurable colour change, whereas erythema was markedly less visible in the darker-skinned cohort.

Asare-Baiden et al. [36] demonstrated that temperature-based changes were detectable across all skin tones, with thermography reliably identifying early tissue alterations under varied lighting conditions and at different anatomical sites. Collectively, these findings suggest that thermography provides a tone-independent method for early detection of pressure injuries by measuring sub-surface infrared emissions rather than reflected surface light. Asare-Baiden et al. [36] conducted a quasi-experimental study involving 35 adults, of whom 30 had darker skin tones and five had lighter skin tones, in order to evaluate the performance of infrared thermography combined with convolutional neural network (CNN) processing (MobileNetV2) in varied positions and at various distances following mechanical loading. The study found that thermography paired with CNN analysis was substantially more reliable than visual inspection alone for detecting early pressure-related tissue changes, particularly in individuals with darker skin tones. Infrared thermography consistently identified early thermal anomalies that were not visible during routine visual assessment.

#### 3.3.2. Subepidermal Moisture (SEM) Sensors

Three studies examined the utilisation of subepidermal moisture (SEM) sensors for early tissue assessment [34,35,38]. Osborne Chambers, and Thompson [34] conducted a quality improvement project involving 140 adult critical care patients across a 24-week period, comprising a 12-week pre-implementation phase (*n* = 90) and a 12-week implementation phase (*n* = 50). Although demographic characteristics were not reported for the full cohort, all eight facility-acquired pressure injuries (HAPIs) identified during the pre-implementation period occurred in African American patients, and 35 African American patients were included in the implementation phase. SEM technology reliably identified early tissue damage across diverse skin tones, including richly pigmented skin, and its integration into routine assessment was associated with a marked reduction in HAPI incidence. Specifically, HAPI rates decreased from 8.9% (8/90) pre-implementation to 0% (0/50) during the SEM implementation period, representing a complete elimination of new injuries. The authors attributed this substantial improvement to earlier detection of tissue compromise and more targeted preventive care enabled by SEM scanning.

Zamarripa [35] found that elevated SEM values successfully predicted pressure injury development across skin tones and preceded visual manifestations. Zamarripa [35] conducted a non-experimental repeated-measures descriptive study involving 122 acute care patients across diverse skin tones to compare subepidermal moisture (SEM) measurements using the Delphin Moisture Meter D with traditional visual assessment and the Braden Scale. The study found that SEM values consistently identified early tissue changes before any visible skin breakdown, demonstrating its utility as an objective early-warning indicator across skin tones. In contrast, visual assessment required an average of 4.3 days to confirm tissue changes, highlighting the substantial delay inherent in surface-level inspection. The study further emphasised that assessing patients with darker skin tones remains a clinical challenge, reinforcing the need for objective, tone-independent technologies to support earlier and more equitable pressure injury detection.

Pittman et al. [38] conducted a retrospective descriptive comparative study involving 69 participants to evaluate the use of long-wave infrared thermography for early pressure injury detection, and 42% of the participants identified as African American. Clinicians at the bedside noted that the SEM device was easy to use, enhanced overall skin assessment accuracy, and bypassed the visual ambiguity common in dark skin tone assessments [38]. The study found that temperature asymmetry reliably preceded visible pressure injury development across all skin tones, demonstrating the value of thermography as an objective early-warning indicator. Subepidermal moisture (SEM) values identified tissue changes 3–10 days before visual signs, offering strong predictive accuracy and highlighting the limitations of traditional surface-level inspection. The authors noted that these objective technologies have the potential to reduce racial disparities in pressure injury detection, particularly by improving early identification in individuals with darker skin tones where visual cues are less apparent.

#### 3.3.3. Image Processing and Colorimetry

Two studies used image processing and colometry to standardise skin assessment. The first was that by Li and Mathews [37], who developed an automated image segmentation algorithm using Support Vector Machines (SVM), achieving high inter-rater reliability for objective wound boundary measurement. SVM operated completely free of human colour bias. Li and Mathews [37] conducted a cross-sectional algorithm development study using 32 clinical pressure injury images to evaluate an automated image processing approach for pressure injury measurement. Their method applied YCbCr color-space segmentation combined with Support Vector Machine (SVM) classification to delineate wound boundaries and quantify tissue damage. The study demonstrated that automated segmentation and measurement achieved higher inter- and intra-rater reliability compared with manual visual assessment. Importantly, the computer vision-generated boundaries provided objective and tone-independent measurements of localised tissue lesions, highlighting the potential of image processing algorithms to support more consistent and equitable pressure injury assessment across diverse skin tones. Studies using Artificial Intelligence did not report any details about how their Support Vector Machine models were trained to avoid algorithmic bias related to skin colour. None described their training datasets or any measures to ensure equitable performance across different skin tones.

Similarly, Sonenblum et al. [33] employed colorimetric devices to detect or map erythema across diverse skin tones. They conducted a pre/post experimental study involving 61 adults across diverse skin tones (3 Hispanic, 16 Asian, 18 Black, 23 White, and 4 individuals identifying with more than one race) to evaluate the performance of multiple colorimetric tools including the ColorMeter DSM III, Munsell and Pantone scales, and eumelanin grouping in detecting induced erythema. The study found that the ColorMeter DSM III effectively measured both skin tone and erythema across all pigmentation groups, offering greater precision than traditional visual assessment methods used in pressure injury detection. The use of melanin-adjusted scales improved accuracy by accounting for baseline pigmentation differences, thereby reducing the subjectivity inherent in conventional nursing skin colour charts. In addition, thermal asymmetry reliably indicated early tissue stress, with patterns remaining consistent across skin tones, further supporting the value of objective, tone-independent technologies for early pressure injury identification.

#### 3.3.4. Enhanced Lighting-Supported Assessment

Two studies discussed enhanced lighting as a strategy in assessment of dark skin tones. Waidley et al. [16] conducted a small-scale quality improvement project using quantitative methods with 10 participants in a unit/ward, of which 40% were of minority racial groups, to evaluate the effectiveness of the Enhanced Skin Assessment for Dark Skin (SADS) technique combined with halogen lighting. The study found that SADS used alongside halogen lighting detected early pressure-related skin changes earlier than standard visual assessment, and this approach was effective across all skin tones. Although the study did not report the exact distribution of skin tones, the authors noted that the SADS technique combined with halogen lighting was effective across skin tones, indicating that participants with darker pigmentation were included. This enhanced protocol incorporated assessment of colour change, pain, firmness or softness, and temperature differences. Implementation of the enhanced assessment protocol resulted in a 6% reduction in facility-acquired pressure injuries, demonstrating the value of structured, tone-inclusive assessment methods in improving early detection and prevention outcomes.

Scafide et al. [39] conducted a descriptive prospective study involving 10 adults, 40% of whom identified with minority racial groups, to evaluate alternate light source (ALS) technology for visual skin assessment. Participants underwent assessment under standard white light, violet ALS (406 nm), blue ALS (448 nm), and ultrasound for non-extremity sites. The study found that ALS revealed sub-surface tissue damage not visible under standard environmental white light, delineating larger apparent pressure injury boundaries than those identified visually. On average, injury areas increased by 17.5 cm under violet light and 13.7 cm under blue wavelengths, demonstrating ALS’s capacity to enhance boundary detection. Importantly, both studies do not report any adverse effects or patient discomfort when alternate light sources were applied.

Across the ten included studies, four categories of assessment strategies were identified: thermographic imaging, subepidermal moisture (SEM) sensors, image processing and colorimetry approaches, and enhanced lighting-supported assessment. Although these strategies differed in technological complexity and measurement principles, a consistent pattern emerged. Objective technologies reliably detected early tissue changes before visual signs appeared and performed effectively across diverse skin tones, addressing a critical diagnostic equity gap. Thermographic imaging demonstrated tone-independent detection of subsurface thermal anomalies that preceded visible injury. SEM sensors consistently identified early tissue changes several days before visual confirmation and contributed to reductions in facility-acquired pressure injuries. Image processing and colorimetric tools provided reproducible, bias-free metrics that improved erythema detection and standardised wound boundary measurement across pigmentation levels. Enhanced lighting approaches improved visibility of tissue changes masked by melanin, with halogen lighting supporting earlier detection and alternate light sources revealing subsurface changes not visible under standard illumination, though benefits were variable for darker skin tones. Collectively, the findings show that routine visual assessment remains insufficient for early pressure injury detection, particularly in dark skin tones, and they highlight the absence of multimodal assessment approaches, representing a key opportunity for future research.

## 4. Discussion

The findings of this scoping review indicate that objective technologies should be systematically integrated into clinical environments to aid in the detection of pressure injuries for individuals with dark skin tones. When the four sub-themes are explored collectively, they describe how different technologies—such as thermal imaging, moisture sensors, digital colour metrics, and better bedside lighting—can work together to offer solutions to a longstanding problem. The tools work differently whilst sharing the same goal: giving clinicians an objective, reliable way to identify early tissue damage without reliance on skin colour. Across all the studies, these methods consistently improved on traditional visual skin checks, identifying injuries days earlier and helping clinicians reach the same accurate conclusion. This literature maps a critical paradigm shift toward technologically enabled assessment frameworks designed to dismantle diagnostic inequities and safeguard richly pigmented skin.

The compiled evidence demonstrates that these tools maintain diagnostic performance across the entire spectrum of human skin pigmentation [32,33,38]. By shifting the clinical paradigm from reliance on subjective surface visualisation to objective biophysical and thermodynamic measurement, these strategies directly address the critical equity gap in early pressure injury identification and prevention. However, the findings also highlight a critical limitation in current clinical practice: the persistent, systemic over-reliance on visual assessment alone [40]. Evidence consistently demonstrates that visual cues such as erythema, blanching, or subtle pink hues are fundamentally unreliable in dark-skin-tone individuals. High melanin concentrations mask standard surface colour modifications, resulting in missed or delayed identification of early-stage tissue damage [14]. These diagnostic challenges contribute directly to inequitable clinical outcomes, placing individuals with dark skin tones at a disproportionately higher risk of progressing to advanced, unstageable, or full-thickness tissue destruction [21].

Technological adjuncts such as thermography, SEM sensors, image processing algorithms, and colorimetry are emerging as vital objective counter-measures to these visual limitations. Studies employing thermographic imaging revealed temperature anomalies indicative of inflammation or ischemia prior to the appearance of visual signs [31,32,36]. SEM technology implemented in both acute and critical care settings consistently detected subclinical tissue changes and enabled timely interventions [34,35,38]. Similarly, image processing and colorimetric approaches offered objective, reproducible metrics for wound measurement and erythema detection across skin tones [33,37]. Importantly all these technologies contributed to improved diagnostic equity by maintaining reliable performance across diverse skin tones. Across the included studies, the strategies demonstrated reliable performance for patients with varying skin tones, underscoring the capacity of objective technologies to address longstanding skin tone-based inequities in pressure injury identification [34].

These findings carry implications for contemporary clinical practice. Collectively, they support the systematic integration of objective technological modalities into routine skin assessment protocols, particularly within acute environments such as intensive care units, where patients experience profound immobility and multi-system physiological compromise [1,2]. However, as formal appraisal of the manuscripts was not undertaken, recommendations for integration should be considered cautiously and be supported by future high-quality evidence. These findings further highlight the need for culturally safe education and training frameworks that enable clinicians to accurately interpret technological outputs alongside contextual patient risk factors [16,38,39].

Another key finding of this review is the striking scarcity of primary research critically evaluating routine, everyday bedside visual and tactile skin assessments within acute care. Despite serving as the frontline clinical method, traditional tactile and visual inspections (and their reliance on problematic cues like erythema and blanching) are heavily underrepresented in the literature concerning dark skin tone [3,13]. This evaluation gap perpetuates systematic blind spots and diagnostic bias. For instance, Lo et al. [40] highlighted that intensive care unit nurses face a profound lack of confidence when conducting these checks on patients with dark skin tones, citing a pervasive culture of ‘white normativity’ where available training tools remain implicitly shaped around light-skin-tone benchmarks. Developing accessible, affordable, and culturally safe manual assessment approaches can help reduce persistent inequities and support care for all patients, regardless of skin tone. While objective diagnostic technologies offer a promising pathway to address these inequities, their high procurement costs and infrastructure demands can restrict widespread adoption in resource-constrained environments [41].

Studies used varied approaches to defining dark skin tone, which limits comparability across findings and is noted as a methodological limitation in the review. All studies included in this scoping review originated from the United States of America and while this provides a coherent contextual backdrop, it also means that the findings may not fully represent experiences or outcomes in other countries. Furthermore, although several of the technologies discussed in the included studies are highly specialized, it is likely that only some will be feasible outside of well-resourced environments. However, simpler approaches such as improved lighting, structured bedside assessment, and focused clinician training remain transferable and practical. A further limitation of this review was the restriction to literature published in English only, which may have led to selection bias and relevant manuscripts not being captured. Consequently, achieving true clinical equity requires a balanced, scalable strategy. Alongside technological innovation, future research must urgently interrogate and reform everyday bedside assessment frameworks. Further, research from diverse international settings would strengthen the evidence base and support broader applicability.

## 5. Conclusions

This scoping review highlights the growing use of objective technological approaches in skin assessment for early pressure injury detection, while simultaneously exposing a critical gap in clinical practice: the lack of evidence examining routine visual and tactile assessment methods. Although technology-based strategies demonstrated value in identifying early tissue damage across diverse skin tones, no studies systematically evaluated the everyday bedside practices that remain central to clinical care.

The findings also show that while objective technologies have the potential to reduce skin tone-based disparities, their cost and limited availability risk reinforcing inequities by restricting access to well-resourced settings. This dual challenge reflects broader systemic injustices that shape patient outcomes. Advancing equity in pressure injury prevention will require research that interrogates routine assessment practices alongside technological innovations, as well as guidelines that embed equity-oriented protocols and culturally safe training responsive to contextual patient factors. Routine visual assessment remains insufficient, highlighting a critical equity gap.

Ultimately, this review calls for a paradigm shift from reactive identification of visible damage to proactive, technology-enhanced detection strategies. Such a shift is essential to achieving justice, improving outcomes across diverse populations, and ensuring that pressure injury prevention is both evidence-based and equitable. Integrating objective technologies into routine practice may improve diagnostic accuracy and equity in acute care.

## Figures and Tables

**Table 1:** Characteristics of the studies.

Author(s), Year	Country	Study Design	Participants	Intervention/Exposure	Key Findings
Asare-Baiden et al., 2024 [36]	USA	Quasi-experimental	35 adults (30 darker skin tones; 5 lighter skin tones)	Infrared thermography and CNN processing (MobileNetV2) under varied positions/distances following mechanical loading.	Thermography and convolutional neural networks were revealed to be more reliable in detecting pressure injury in darker skin tones than visual inspection alone. Thermography detected early changes more reliably than visual assessment.
Bates-Jensen, et al., 2024 [32]	USA	Pre/post experimental methods study	61 participants (18 African American or Black; 16 Asian American, 23 White; 4 mixed race)	Thermal measurement of erythema across skin tones	Thermal patterns were consistent across tones, proving infrared emissivity’s tone-independence, while erythema visibility varied significantly by skin tone. The lighter skin tones demonstrated higher measured changes compared to the darker-skin cohort.
Lam, 2020 [31]	USA	Case studies	3 patients with dark skin tones at risk of developing PI	Long-wave infrared thermography (LWIT) and digital photography	Thermal anomalies were observed before visual signs of PI appeared. LWIT was effective in early detection of PI.
Li & Mathews, 2017 [37]	USA	Cross-sectional algorithm development study	32 participants (clinical pressure injury images)	Image processing algorithm for automated PI measurement using YCbCr colour spaces and Support Vector Machines.	Automated segmentation/measurement achieved higher inter-/intra-rater reliability. Computer vision boundaries provided objective and independent measurements of localised tissue lesions.
Osborne Chambers & Thompson, 2024 [34]	USA	Quality improvement using quantitative methods	140 participants—Adults/diverse skin tones. 25% of participants were African American	Subepidermal moisture (SEM) sensor.	SEM technology detected early-stage tissue damage across skin tones, resulting in significant reductions in PI during the study.
Pittman et al., 2024 [38]	USA	Retrospective, descriptive comparative study	69 participants	Longwave infrared Thermography	Detected enabled temperature asymmetry preceding visible pressure injury development across skin tones SEM identified tissue changes 3–10 days before visual signs, with strong predictive accuracy; this would support reducing racial disparities.
Scafide et al., 2025 [39]	USA	Descriptive, prospective study	10 adults (40% minority racial groups)	Alternate light source(ALS) visual skin assessment conducted under: White light (standard care) Violet ALS (406 nm) Blue ALS (448 nm) Ultrasound (for non-extremity sites)	ALS assessment revealed sub-surface tissue damage not visible under standard environmental white light. ALS delineated larger apparent PI boundaries areas than white light. The mean increase in identified injury areas was +17.5 cm under violet, 13.7 cm under blue wavelengths. ALS improved erythema visibility in lighter skin; there was limited benefit in darker skin, and color-dependent limitations persisted.
Sonenblum et al., 2023 [33]	USA	Pre/post experimental study	61 adults (3 Hispanic; 16 Asian; 18 Black; 23 White; 4 identified with more than one race)	ColorMeter DSM III, Munsell, Pantone, Eumelanin grouping; induced erythema	ColorMeter effectively measured skin tone and erythema across tones, improving on the current standards for detection of PI. Thermal asymmetry reliably indicated early tissue stress; patterns were consistent across skin tones.
Waidley et al., 2024 [16]	USA	Quality improvement project using quantitative methods	10 participants	Enhanced skin assessment for dark skin (SADS) + halogen lighting.	Use of SADS + halogen lighting predicted PI development earlier than visual assessment and was effective across skin tones. It achieved a 6% decrease in facilities acquired PIs.
Zamarripa, 2021 [35]	USA	Nonexperimental repeated-measures descriptive design	122 acute care patients across diverse skin tones	Delphin Moisture Meter D dermal phase meter (DPM) vs. Braden scale and visual checks.	SEM measurements assisted in early detection before skin surface breakdown. Visual assessment took 4.3 days to confirm changes and assessing patients with skin tones remains a challenge.

## Data Availability

No new data were created or analyzed in this study. Data sharing is not applicable to this article.

## Appendix A

PubMed

((“Skin Pigmentation”[Mesh]) OR (“higher melanin”[tw] OR “ethnic skin”[tw] OR “skin colour”[tw] OR “skin color”[tw] OR “skin tone*”[tw] OR “dark skin”[tw] OR “darker skin”[tw] OR “skin pigmentation*”[tw] OR “diverse skin”[tw] OR Ethnicity[tw] OR race[tw])) AND ((“Pressure Ulcer”[Mesh]) OR (“pressure injur*”[tw] OR “bed sore*”[tw] OR Bedsore*[tw] OR “decubitus ulcer*”[tw] OR “decubitus sore*”[tw] OR “pressure ulcer*”[tw]))

CINAHL (Ebsco)

(((MH “Skin Pigmentation+”)) OR (“higher melanin” OR “ethnic skin” OR “skin colour” OR “skin color” OR “skin tone*” OR “dark skin” OR “darker skin” OR “skin pigmentation*” OR “diverse skin” OR Ethnicity OR race)) AND (((MH “Pressure Ulcer+”)) OR (“pressure injur*” OR “bed sore*” OR Bedsore* OR “decubitus ulcer*” OR “decubitus sore*” OR “pressure ulcer*”))

Scopus

((“Skin Pigmentation”) OR (“higher melanin” OR “ethnic skin” OR “skin colour” OR “skin color” OR “skin tone*” OR “dark skin” OR “darker skin” OR “skin pigmentation*” OR “diverse skin” OR Ethnicity OR race)) AND ((“Pressure Ulcer”) OR (“pressure injur*” OR “bed sore*” OR Bedsore* OR “decubitus ulcer*” OR “decubitus sore*” OR “pressure ulcer*”))

Web of Science

((“Skin Pigmentation”) OR (“higher melanin” OR “ethnic skin” OR “skin colour” OR “skin color” OR “skin tone*” OR “dark skin” OR “darker skin” OR “skin pigmentation*” OR “diverse skin” OR Ethnicity OR race)) AND ((“Pressure Ulcer”) OR (“pressure injur*” OR “bed sore*” OR Bedsore* OR “decubitus ulcer*” OR “decubitus sore*” OR “pressure ulcer*”))

Google Scholar

(“skin tone” OR “dark skin” OR “darker skin” OR “skin colour” OR “skin color”

OR “skin pigmentation” OR “ethnic skin” OR “higher melanin” OR ethnicity OR race)

AND (“pressure injury” OR “pressure ulcer” OR “bed sore” OR “decubitus ulcer”)

Note

In consultation with the research librarian, Concepts 3, 4, and 5 were excluded from the final search strategy because their inclusion substantially restricted the number of retrieved records and risked omitting relevant studies. These were considered manually by reviewers at all stages of screening.

**Table A1:** Preferred Reporting Items for Systematic reviews and Meta-Analyses extension for Scoping Reviews (PRISMA-ScR) Checklist.

Section	Item	PRISMA-ScR Checklist Item	Reported on Page #
**Title**			
Title	1	Identify the report as a scoping review.	Page 1
**Abstract**			
Structured summary	2	Provide a structured summary that includes (as applicable): background, objectives, eligibility criteria, sources of evidence, charting methods, results, and conclusions that relate to the review questions and objectives.	Page 3–4
**Introduction**			
Rationale	3	Describe the rationale for the review in the context of what is already known. Explain why the review questions/objectives lend themselves to a scoping review approach.	Page 6–7
Objectives	4	Provide an explicit statement of the questions and objectives being addressed with reference to their key elements (e.g., population or participants, concepts, and context) or other relevant key elements used to conceptualize the review questions and/or objectives.	Page 6–7
**Methods**			
Protocol and registration	5	Indicate whether a review protocol exists; state if and where it can be accessed (e.g., a Web address); and if available, provide registration information, including the registration number.	Page 1
Eligibility criteria	6	Specify characteristics of the sources of evidence used as eligibility criteria (e.g., years considered, language, and publication status) and provide a rationale.	Page 8–9
Information sources *	7	Describe all information sources in the search (e.g., databases with dates of coverage and contact with authors to identify additional sources), as well as the date the most recent search was executed.	Page 9
Search	8	Present the full electronic search strategy for at least 1 database, including any limits used, such that it could be repeated.	Page 9
Selection of sources of evidence †	9	State the process for selecting sources of evidence (i.e., screening and eligibility) included in the scoping review.	Page 10
Data charting process ‡	10	Describe the methods of charting data from the included sources of evidence (e.g., calibrated forms or forms that have been tested by the team before their use, and whether data charting was done independently or in duplicate) and any processes for obtaining and confirming data from investigators.	Page 10–11
Data items	11	List and define all variables for which data were sought and any assumptions and simplifications made.	Page 11–12
Critical appraisal of individual sources of evidence §	12	If done, provide a rationale for conducting a critical appraisal of included sources of evidence; describe the methods used and how this information was used in any data synthesis (if appropriate).	N/A
Synthesis of results	13	Describe the methods of handling and summarizing the data that were charted.	Page 12
**Results**			
Selection of sources of evidence	14	Give numbers of sources of evidence screened, assessed for eligibility, and included in the review, with reasons for exclusions at each stage, ideally using a flow diagram.	Page 11
Characteristics of sources of evidence	15	For each source of evidence, present characteristics for which data were charted and provide the citations.	Page 12
Critical appraisal within sources of evidence	16	If done, present data on critical appraisal of included sources of evidence (see item 12).	N/A
Results of individual sources of evidence	17	For each included source of evidence, present the relevant data that were charted that relate to the review questions and objectives.	Page 12
Synthesis of results	18	Summarize and/or present the charting results as they relate to the review questions and objectives.	Page 12–18
**Discussion**			
Summary of evidence	19	Summarize the main results (including an overview of concepts, themes, and types of evidence available), link to the review questions and objectives, and consider the relevance to key groups.	Page 18–20
Limitations	20	Discuss the limitations of the scoping review process.	Page 20
Conclusions	21	Provide a general interpretation of the results with respect to the review questions and objectives, as well as potential implications and/or next steps.	Page 20
**Funding**			
Funding	22	Describe sources of funding for the included sources of evidence, as well as sources of funding for the scoping review. Describe the role of the funders of the scoping review.	N/A

# = number; JBI = Joanna Briggs Institute; PRISMA-ScR = Preferred Reporting Items for Systematic reviews and Meta-Analyses extension for Scoping Reviews. * Where sources of evidence (see second footnote) are compiled from, such as bibliographic databases, social media platforms, and Web sites. † A more inclusive/heterogeneous term used to account for the different types of evidence or data sources (e.g., quantitative and/or qualitative research, expert opinion, and policy documents) that may be eligible in a scoping review as opposed to only studies. This is not to be confused with information sources (see first footnote). ‡ The frameworks by Arksey and O’Malley (6) and Levac and colleagues (7) and the JBI guidance (4, 5) refer to the process of data extraction in a scoping review as data charting. § The process of systematically examining research evidence to assess its validity, results, and relevance before using it to inform a decision. This term is used for items 12 and 19 instead of “risk of bias” (which is more applicable to systematic reviews of interventions) to include and acknowledge the various sources of evidence that may be used in a scoping review (e.g., quantitative and/or qualitative research, expert opinion, and policy document). From: Tricco AC, et al., 2018 [42].
